# Effect of Transcutaneous Vagus Nerve Stimulation at Auricular Concha for Insomnia: A Randomized Clinical Trial

**DOI:** 10.1155/2020/6049891

**Published:** 2020-08-07

**Authors:** Yue Jiao, Xiao Guo, Man Luo, Suxia Li, Aihua Liu, Yufeng Zhao, Bin Zhao, Dequan Wang, Zaifang Li, Xiaojiao Zheng, Mozheng Wu, Peijing Rong

**Affiliations:** ^1^Institute of Acupuncture and Moxibustion, China Academy of Chinese Medical Sciences, Beijing 100700, China; ^2^National Institute on Drug Dependence, Peking University, Beijing 100191, China; ^3^Neurology Department, Xuanwu Hospital of Capital Medical University, Beijing 100053, China; ^4^Chinese Medicine Data Center, China Academy of Chinese Medical Sciences, Beijing 100700, China

## Abstract

Insomnia inflicts mental burden and decreases physical productivity and affects life quality. Transcutaneous vagus nerve stimulation (ta-VNS) may be an effective treatment option for insomnia. This study aims to evaluate the effect and safety of ta-VNS and compare it with transcutaneous nonvagus nerve stimulation (tn-VNS). A multicenter, randomized, clinical trial was conducted at 3 hospitals in China enrolling 72 insomnia participants from May 2016 to June 2017. Participants were randomly assigned (1 : 1) to receive 40 sessions of ta-VNS or tn-VNS treatment. 63 participants completed the trial. ta-VNS treatment significantly decreased the Pittsburgh Sleep Quality Index score, Epworth Sleepiness Scale score, Flinders Fatigue Scale score, Hamilton Depression Scale score, and Hamilton Anxiety Scale score over 4 weeks compared with those of the baseline. Moreover, it also significantly increased the 36-item Short-Form Health Survey Questionnaire scores compared with that of the baseline. However, it did not show significant differences compared with tn-VNS in changes of primary and secondary outcomes. The incidence of adverse events was low. ta-VNS significantly relieved insomnia over 4 weeks. Moreover, it also alleviated fatigue and improved participants' quality of life as well as other concomitant symptoms such as depression and anxiety. This trial is registered at Chinese Clinical Trial Registry (http://www.chictr.org.cn) with the registration number: ChiCTR-TRC-13003519.

## 1. Introduction

Insomnia is a sleep-wake disorder commonly seen in the clinic that includes difficulty initiating sleep, difficulty maintaining sleep, or early-morning awakening with the inability to return to sleep, resulting in daytime impairment [1]. About 30% to 35% of people around the world have symptoms of sleep difficulties, and the prevalence is higher among women [1, 2]. Long-term insomnia relates to body impairment, multiple organ dysfunction, and immune decline, as well as depression, anxiety, and other mental diseases [3]. It inflicts mental burden, decreases physical productivity, and affects life quality on individuals and the broader community. However, due to treatment and management barriers, insomnia often remains unrecognized and untreated.

Transcutaneous vagus nerve stimulation (ta-VNS) may be an effective treatment option for insomnia. ta-VNS is a noninvasive neurostimulation technique applied to the afferent auricular branch of the vagus nerve in the auricular concha, especially cymba concha [4]. Currently, invasive VNS is approved by the U. S. Food and Drug Administration (FDA) to treat refractory epilepsy and depression [5]. Two ta-VNS trials were conducted involving patients with intractable epilepsy and major depression [6–8]. According to them, we found insomnia as a concomitant symptom in some patients who were also relieved. Insomnia often co-occurs with depression, and they also share a neural basis that some increased functional connectivity between the same brain regions [9]. And, sleep disorders are also common in patients with epilepsy; seizure patterns can be affected by sleep cycles and vice versa [10]. From the above, we assumed that ta-VNS might also be a choice for insomnia. Furthermore, insomnia increased the activity of the autonomic nervous system [2]. Vagus nerve stimulation might balance the hyperactivity of the sympathetic nerve. Last but not least, auricular acupuncture at auricular concha, where the vagus nerve is distributed, appeared to be effective in treating insomnia in previous studies [11].

In this study, we applied transcutaneous nonvagus nerve stimulation (tn-VNS) as a sham comparator. The main objective of this study was to evaluate the effect and safety of ta-VNS and compare it with tn-VNS in improving the sleep quality of participants with insomnia.

## 2. Materials and Methods

### 2.1. Study Design

This randomized, double-blinded, controlled clinical study was conducted from May 2016 to June 2017. The Ethics Committee of Acupuncture and Moxibustion Institute of China Academy of Chinese Medical Sciences (CACMS) approved the study. Written informed consents were obtained from all participants.

### 2.2. Setting

This multicenter study was carried out at 3 settings in China. They were Acupuncture and Moxibustion Hospital of CACMS, Xuanwu Hospital of Capital Medical University, and National Institute on Drug Dependence of Peking University, respectively.

### 2.3. Participants

Participants with insomnia disorder were recruited from hospitals via posters or physician referrals. Participants were eligible if they were 18∼70 years old, diagnosed as insomnia disorder according to the Diagnostic and Statistical Manual of Mental Disorders, Fifth Edition (DSM-5), and voluntary to participate. The exclusion criteria were pregnancy, comorbidity of severe primary diseases from the heart, brain, liver, kidney and hemopoietic system, inadequate sleep hygiene, and related drug use. Participants were compensated for study participation. After the study, each participant was given a ta-VNS apparatus with the ear-clip design.

### 2.4. Interventions

#### 2.4.1. ta-VNS Treatment

**Figure 1:**
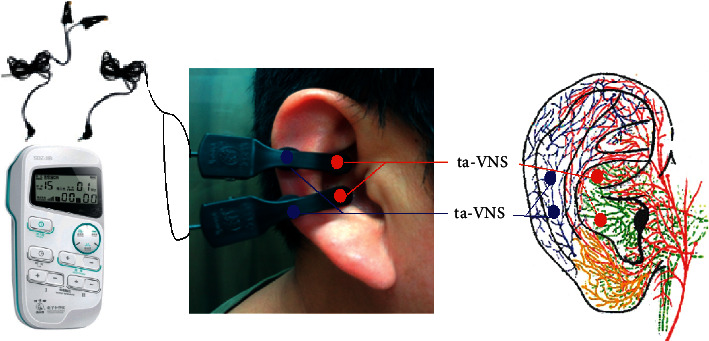
Stimulating regions of ta-VNS and tn-VNS. Red dots indicate regions of ta-VNS (ABVN: green color); blue dots indicate regions of tnVNS (LON: blue color).

*(1) Stimulating Region*. Auricular concha innervated by afferent auricular branch of the vagus nerve (ABVN) (Figure 1).

*(2) Intervention*. Participants took the supine or sitting position during treatment. After skin disinfection, transcutaneous electrostimulation was applied to the bilateral auricular concha with two pairs of electrode clips via the electric stimulator (Hwato brand SDZ-IIB, Suzhou, Jiangsu, China). The electrodes made of conductive rubber were shaped into ear clips, with a diameter of 5 mm (Figure 1).

The electrical stimulation persisted for 30 minutes with a dilatational wave of 20 Hz (20 Hz for 10 s, 4 Hz for 5 s, and the frequency ratio of sparse wave and the dense wave is 1 : 5), and intensity was adjusted by participants. Participants received 2 treatment sessions per day, one in the morning and the other in the evening. Thirty minutes before bedtime was recommended. Treatment lasted for 5 consecutive days a week, 2 days for rest, and 4 weeks in total.

#### 2.4.2. tn-VNS Group

*(1) Stimulating Region*. Scapha of the auricle innervated not by auricular branch of the vagus nerve (ABVN) but by the lesser occipital nerve (LON) [12, 13] (Figure 1).

*(2) Intervention*. After skin disinfection, transcutaneous electrostimulation was applied to the bilateral auricular scapha with two pairs of electrode clips. The other treatment procedures were the same as those in the ta-VNS group (Figure 1).

Participants were taught to operate the electric stimulator and did treatments at home [14]. They were also asked to document the treatment time in a dairy.

### 2.5. Outcomes

The primary outcome was the change from baseline in the Pittsburgh Sleep Quality Index (PSQI) score to 4 weeks [15]. It is a self-report questionnaire assessing sleep quality for one month that includes 7 components: sleep quality, sleep latency, sleep duration, sleep efficiency, sleep disturbance, hypnotic use, and daytime dysfunction.

The secondary outcomes included the following: the change from baseline in the Epworth Sleepiness Scale (ESS) score (range, 0∼24, with higher scores indicating more sleepiness at daytime; higher than 11 points expressing over sleepiness) to 4 weeks [16]; the change from baseline in the Flinders Fatigue Scale (FFS) score (range, 0∼31, with higher scores indicating more fatigue) to 4 weeks [17]; the change from baseline in the 17-item Hamilton Depression Scale (HAMD) score (range, 0∼51, with higher scores indicating more depression; 7 as minimal clinically important differences) to 4 weeks [18]; the change from baseline in the 17-item Hamilton Anxiety Scale (HAMA) score (range, 0∼56, with higher scores indicating more anxiety; 7 as minimal clinically important differences) to 4 weeks [19]; changes from baseline in the 36-item Short-Form Health Survey Questionnaire (SF-36) scores from 8 sections, including physical functioning (P. F.), role physical (R. P.), bodily pain (B. P.), general health (G. H.), vitality (V. T.), social functioning (S. F.), role emotional (R. E.), mental health (M. H.), lower scores indicating more disability to 4 weeks [20]. And, the severity range of depression was also evaluated by HAMD. A score of 0∼7 is generally accepted to be within the normal range (or in clinical remission), while a score between 8 ∼17 is considered to be mild depression, a score between 18∼23 indicates at least moderate severity, and a score of 24 or higher indicates severe depression [21]. The severity range of anxiety is also evaluated by HAMA. A score of 0∼7 is generally accepted to be within the normal range (or in clinical remission), while a score between 8∼14 may be considered possible anxiety, a score between 15∼21 indicates positive anxiety, a score between 22∼29 indicates obvious anxiety, and a score of 24 or higher indicates severe depression [21]; safety indexes included blood pressure, electrocardiogram analysis, respiratory rate, pulse rate, blood routine, blood biochemistry, and urine analysis. Adverse events were documented via sleep diary during treatment. All participants were required to take a sleep diary, which was collected every week to evaluate the adverse events.

### 2.6. Statistical Analysis

Since no previous study has applied ta-VNS as the single treatment for insomnia, as a novel treatment, we enrolled 30 participants for each group (referring to the requirement of China Food and Drug Administration: at least 20–30 cases in each group in phase I clinical drug trial), to compensate for a 20% loss. The sample size was increased to 36 participants in each group and 72 in total.

All outcomes, including scores of PSQI, ESS, FFS, HAMD-17, HAMA-17, and SF-36, were measured at week 0 and week 4. Our analyses were based on the P. P. principle. All statistical analyses were performed by using SAS version 9.4 (SAS Institute Inc) with a 2-sided *P* value <0.05 considered significant. For continuous variables, comparisons between treatment groups were assessed by using the *t* test or Wilcoxon rank-sum test as appropriate. Categorical variables were compared by using the chi-squared test or Fisher exact test as appropriate.

A sensitivity analysis also was performed by using a mixed-effect model with a repeated measure approach. The model included change from baseline to 4-week as response variables, fixed-effects factors for treatment, visit, treatment × visit interaction, random-effects factors for the patient, site, and site × treatment interaction. An unstructured correlation matrix was used to model the within-patient errors. Parameters were estimated by using the maximum likelihood.

## 3. Results

**Figure 2:**
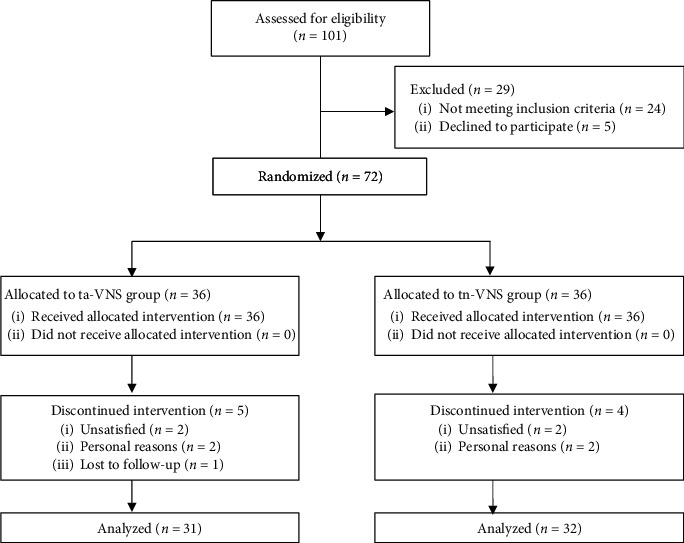
Trial flow diagram.

rom May 2016 to June 2017, 72 participants were randomly assigned either to the ta-VNS group (*n* = 36) or tn-VNS group (*n* = 36) with mean (S. D.) age of 48.78 (11.97) years. Among them, 30 were allocated at Xuanwu Hospital, 31 at National Institute on Drug Dependence, and 11 at Acupuncture and Moxibustion Hospital. Among the randomized participants, 63 completed the study (Figure 2).

Baseline characteristics were similar between the two groups except for HAMA (Table 1).

For the primary outcome, the mean PSQI score was 14.5 (95% CI, 9.0–20.0) points at baseline and 8.8 (95% CI, 4.1–14.0) at week 4 in the ta-VNS group. In the tn-VNS group, the score was 14.5 (95% CI, 10.0–18.7) at baseline and 10.0 (95% CI, 5.0–18.9) at week 4 (Table 2). PSQI scores were superior at week 4 to baseline significantly in both groups (*P* < 0.001) (Table 2). The reduction of the PSQI score was greater in the ta-VNS group (mean, −5.7) than that in the tn-VNS group (mean, −4.6) at week 4 with a mean difference of −1.8 (95%CI,−3.3–0.9; *P* > 0.05). No differences were found between the 2 groups in the reduction of the PSQI score.

The 7 components of PSQI were analyzed separately. The mean scores of sleep quality, sleep latency, sleep duration, hypnotic use, and daytime dysfunction were superior at week 4 to baseline significantly in both groups (*P* < 0.01). The mean scores of sleep efficiency and sleep disturbance in the ta-VNS group were significantly lower at week 4 than at baseline (*P* < 0.05), but in the tn-VNS group, no differences were found (*P* > 0.05). Score reductions of 7 components in the ta-VNS group at week 4 to baseline all showed no significant differences compared with those in the tn-VNS group (*P* > 0.05) (see Table 2).

For the secondary outcomes, the mean ESS score was 5.9 (95% CI, 0.0–13.0) at baseline and 4.4 (95% CI, 0.0–10.0) at week 4 in the ta-VNS group. In the tn-VNS group, the scores were 6.6 (95% CI, 0.0–15.7) at baseline and 4.6 (95% CI, 0.0–10.0) at week 4, respectively. The reduction of the ESS score was greater in the tn-VNS group than that in the ta-VNS group at week 4 with a mean difference of 0.4 (95% CI, −1.4–2.2; *P* > 0.05), but no differences were found between the 2 groups (Table 2).

The mean FFS score was 14.4 (95% CI, 6.6–23.0) at baseline and 10.9 (95% CI, 4.1–19.9) at week 4 in the ta-VNS group. In the tn-VNS group, the scores were 14.4 (95% CI, 9.0–24.8) at baseline and 11.1 (95% CI, 5.1–18.9) at week 4, respectively. FFS scores were superior at week 4 to baseline significantly in both groups (*P* < 0.01). The reduction of the FFS score was greater in the ta-VNS group than that in the tn-VNS group at week 4 with a mean difference of 0.3 (95% CI, −2.1–2.7; *P* > 0.05), but no differences were found between the 2 groups (Table 2).

The mean SF36-total score was 502.2 (95% CI, 306.5–687.2) at baseline and 576.8 (95% CI, 417.2 to 730.8) at week 4 in the ta-VNS group. In the tn-VNS group, the scores were 441.7 (95% CI, 209.4–625.7) at baseline and 520.8 (95% CI, 290.0–728.9) at week 4, respectively. The increase in SF36-total scores was superior at week 4 to baseline significantly in both groups (*P* < 0.05). And, the score increase was greater in the tn-VNS group than that in the ta-VNS group at week 4 with a mean difference of −17.6 (95% CI, −70.2–35.0; *P* > 0.05), but no differences were found between the 2 groups. The 8 components of SF36 were analyzed separately. The mean general health, vitality, role emotional, and mental health scores were superior at week 4 to baseline significantly in both groups (*P* < 0.05). For scores of physical functioning, role physical, bodily pain, and social functioning, no differences were found between baseline and at week 4 in both groups (*P* > 0.05) (Table 2).

Score reductions of 8 components in the ta-VNS group at week 4 to baseline all showed no significant differences compared with those in the tn-VNS group (*P* > 0.05).

The mean HAMD score was 11.9 (95% CI, 5.0–21.9) at baseline and 6.1 (95% CI, 2.9–11) at week 4 in the ta-VNS group. In the tn-VNS group, the scores were 15.3 (95% CI, 7.6–26.4) at baseline and 7.6 (95% CI, 1.0–18.0) at week 4, respectively. HAMD scores were superior at week 4 to baseline significantly in both groups (*P* < 0.001). The reduction in the HAMD score was greater in the tn-VNS group than that in the ta-VNS group at Week 4 with a mean difference of 1.9 (95% CI, −1.1–4.9; *P* > 0.05), but no differences were found between the 2 groups (Table 2).

There were 29 participants without depression, 6 with mild or moderate depression and 1 with severe depression at baseline in the ta-VNS group, and results showed 31 participants without depression at week 4. There were 24 participants without depression, 7 with mild or moderate depression and 5 with severe depression at baseline in the tn-VNS group, and results showed 29 participants without depression and 3 with mild or moderate depression at week 4.

The mean HAMA score was 11.1 (95% CI, 3.6–22.1) at baseline and 5.4 (95% CI, 0.0–11.0) at week 4 in the ta-VNS group. In the tn-VNS group, the scores were 14.3 (95% CI, 5.3–26.1) at baseline and 6.3 (95% CI, 0.1–15.0) at week 4, respectively. HAMA scores were superior at week 4 to baseline significantly in both groups (*P* < 0.001). The reduction in the HAMA score was greater in the tn-VNS group than that in the ta-VNS group at week 4 with a mean difference of 2.4 (95% CI, −0.2–5.1; *P* > 0.05), but no differences were found between the 2 groups (Table 2).

There were 8 participants without anxiety, 21 with mild anxiety, 5 with moderate anxiety, and 2 with severe anxiety at baseline in the ta-VNS group, and results showed 26 participants without anxiety, 4 with mild anxiety, and 1 with moderate anxiety at week 4. There were 6 patients without anxiety, 13 with mild anxiety, and 10 with moderate anxiety at baseline in the tn-VNS group, and results showed 21 participants without anxiety, 8 with mild anxiety, and 3 with moderate anxiety at week 4.

As for safety indexes, physical examination showed no differences at baseline and week 4. Based on participants' sleep diaries, the main side effect was ear pain due to clamping of electrode clips (2 in the ta-VNS group and 1 in the tn-VNS group). And, other side effects included headache (1 in the ta-VNS group) and slight head fullness (1 in the ta-VNS group). All participants recovered fully from the side events after stopping the treatment.

## 4. Discussion

In this study, we evaluated the therapeutic effect and safety of ta-VNS treatment on participants with insomnia. We found that ta-VNS significantly alleviated insomnia over a 4-week treatment period. Moreover, it also ameliorated fatigue and participants' quality of lifeas well as other concomitant symptoms as depression and anxiety. However, it did not show a significant difference compared with tn-VNS in changes of primary and secondary outcomes. The incidence of adverse events was low.

Similar to our previous study, 35 insomnia patients adopted ta-VNS treatment with a decreased PSQI score of 3.3 at Week 4 [22]. In this study, the decrease in the PSQI score in the ta-VNS group was 5.7 at week 4, and it was superior at week 4 to baseline significantly (*P* < 0.001). And, the mean scores of 7 components of PSQI, sleep quality, sleep latency, sleep duration, hypnotic use, sleep efficiency, sleep disturbance, and daytime dysfunction, were all superior at week 4 to baseline significantly (*P* < 0.01). Thus, the results showed that ta-VNS had a clinically meaningful benefit in relieving insomnia.

ESS and FFS are also important assessments for evaluating insomnia. In this study, the mean score of ESS was not significantly different from week 4 to baseline. It showed that the daytime sleepiness of participants was not improved obviously after treatment. However, FFS scores were superior at week 4 to baseline significantly, and it showed that ta-VNS could alleviate fatigue obviously (*P* < 0.01).

SF-36 is a very widely used scale in evaluating health-related quality of life in insomnia [23]. The increase in SF36-total score of the ta-VNS group was superior at week 4 to baseline significantly (*P* < 0.05). It showed that ta-VNS had a clinically meaningful benefit in improving the quality of life for insomnia participants. In terms of the 8 components, the mean scores of general health, vitality, role emotional, and mental health scores were superior at week 4 to baseline significantly (*P* < 0.05). The results indicated that ta-VNS improved the mental health and subjective feeling towards the life quality of participants.

Depression and anxiety often accompany insomnia. People with insomnia had greater depression and anxiety levels and were 9.82 and 17.35 times likely to have clinically significant depression and anxiety, respectively [24]. Similar to our previous study, 35 insomnia patients adopting ta-VNS treatment got a decreased HAMD score of 3.8 and HAMA score of 5.5 at Week 4 [22]. And, in this study, the decreased HAMD score in the ta-VNS group was 5.8 and the decreased HAMA score was 5.6 at week 4. They were both superior at week 4 to baseline significantly (*P* < 0.001). The results demonstrated that ta-VNS had clinically meaningful benefits in relieving depression and anxiety.

However, ta-VNS treatment had no significant differences compared with the tn-VNS group in relieving insomnia, depression, and anxiety and improving QOL (*P* > 0.05). On the one hand, the probable reason might lie in the electrodes clip on both concha and scapha of the auricle. Therefore, although the electrical stimulation was applied differently, the mechanical force was the same in both groups. On the other hand, ta-VNS treatment for 4 weeks might not be long enough to show a significant difference in relieving insomnia compared with the tn-VNS group.

The mechanism of ta-VNS treatment for insomnia remains unclear. Insomnia is caused by dysregulation of the arousal system, cognitive system, and HPA axis, involving broad encephalic regions, such as the hypothalamus, pineal gland, amygdala, and ventral tegmental area [2, 25]. And, it is very sensitive to the neurotransmitters change, such as the decline of central melatonin [26]. The ta-VNS stimulates the afferent auricular branches of the vagus nerve, that project to the solitary nucleus and then to the limbic and the autonomic nervous system structures, including the pineal gland, ventral tegmental area, hypothalamus, amygdala, anterior cingulate cortex, nucleus accumbens, and lateral prefrontal cortex [27]. Moreover, the ta-VNS triggers a tidal release of melatonin and enhances its production [28]. Thus, ta-VNS treatment probably regulates neural circuits that govern sleep and melatonin secretion to alleviate insomnia.

In a previous study in patients with primary insomnia, Luo et al. [22] found that, compared with the PSQI before treatment, the PSQI significantly decreased at the end of the 2nd week (*P* < 0.05). However, the lack of a control group limited the interpretation. In this study, we designed a control group (tn-VNS) with a double-blinded method, which could enhance the quality of the study.

Of course, this study has several limitations. Firstly, no follow-up was set up in the study. Secondly, we did not design the blank control or positive control group, because it was our first study applying ta-VNS to treat insomnia. The purpose was to compare the therapeutic effects of ta-VNS and tn-VNS. Although there were no significant differences between the two groups, the results showed the efficacy of ta-VNS in treating insomnia. In future studies, we will consider designing a positive control group to confirm its effectiveness. Thirdly, we did not do multiple imputation analyses for all outcomes. Fourthly, the participant blinding assessment was not applied. We will consider all of the above in future design.

## 5. Conclusion

ta-VNS significantly relieved insomnia over a 4-week treatment period. Moreover, it also ameliorated fatigue and participants' quality of life as well as other concomitant symptoms such as depression and anxiety. However, it did not show a significant difference compared with tn-VNS in changes of primary and secondary outcomes.

## Figures and Tables

**Table 1:** Participant baseline characteristics^a^.

Characteristics	ta-VNS (*n* = 36)	tn-VNS (*n* = 36)
Age, mean (S. D.), y	47.67 (12.0)	50.6 (12.0)
Gender		
Male	5 (13.9)	7 (19.4)
Female	31 (86.1)	29 (80.6)
Race		
Han	35 (97.2)	33 (91.7)
Minorities	1 (2.8)	3 (8.3)
Marriage		
Married	30 (83.3)	33 (91.7)
Unmarried	5 (13.9)	3 (8.3)
Divorced	1 (2.8)	0 (0)
Occupation		
Retired or unemployed	8 (22.2)	10 (27.8)
Teacher	6 (16.7)	7 (19.4)
Employee	6 (16.7)	4 (11.1)
Others	16 (44.4)	15 (41.7)
PSQI score, mean (S. D.)^b^	14.5 (3.2)	14.5 (2.7)
Sleep quality	0.8 (1.1)	1.4 (1.4)
Sleep latency	2.5 (0.7)	2.6 (0.7)
Sleep duration	2.0 (1.0)	2.1 (0.9)
Sleep efficiency	1.7 (1.2)	1.4 (1.1)
Sleep disturbances	2.4 (0.5)	2.4 (0.5)
Hypnotic use	2.4 (0.9)	2.1 (1.1)
Daytime dysfunction	2.7 (0.5)	2.5 (0.7)
ESS score, mean (S. D.)	5.9 (4.0)	6.6 (5.0)
FSS score, mean (S. D.)	14.4 (5.2)	14.4 (4.6)
HAMD score, mean (S. D.)	11.9 (5.5)	15.3 (5.6)
HAMA score, mean (S. D.)	11.1 (5.3)	14.3 (6.6)
SF-36 score, mean (S. D.)^c^	502.2 (121.9)	441.7 (133.0)
Physical function	86.3 (12.8)	83.3 (14.9)
Role physical	62.5 (41.2)	44.4 (44.8)
Bodily pain	55.6 (42.9)	36.3 (40.9)
General health	59.7 (19.0)	88.5 (19.6)
Vitality	57.4 (18.6)	52.3 (18.4)
Social functioning	61.0 (24.3)	59.4 (22.4)
Role emotional	73.6 (19.5)	74.9 (21.9)
Mental health	46.1 (17.3)	37.4 (20.2)

^a^Values reported as no. (%) unless otherwise indicated. ^b^Total scores of PSQI, and below 7 components of PSQI are analyzed separately. ^c^Total scores of SF-36, and below 8 components of SF-36 are analyzed separately.

**Table 2:** Primary and secondary outcome comparison between groups.

Variable	ta-VNS (*n* = 31)	tn-VNS (*n* = 32)	Differences	*P* value^a^
Primary outcome^b^				
PSQI score at wk4, mean (95% CI)	8.8 (4.1 to 14.0)	10.0 (5.0 to 18.9)	−1.2 (−3.1 to 0.8)	0.25
Change at wk 4, PSQI score, mean (95% CI)	−5.7 (−7.5 to −4.0)	−4.6 (−6.4 to 2.8)	−1.8 (−3.3 to 0.9)	0.27
Sleep quality^c^	−0.7 (−1.1 to −0.3)	−0.8 (−1.4 to −0.2)	0.1 (−0.4 to 0.7)	0.65
Sleep latency^c^	−0.9 (−1.3 to −0.5)	−0.8 (−1.2 to −0.4)	−0.1 (−0.5 to 0.3)	0.66
Sleep duration^c^	−0.7 (−1.2 to −0.3)	−0.8 (−1.2 to −0.3)	0 (−0.5 to 0.5)	0.98
Sleep efficiency^c^	−0.9 (−1.4 to −0.3)	−0.2 (−0.8 to 0.3)	−0.7 (−1.3 to 0.0)	0.06
Sleep disturbances^c^	−0.3 (−0.5 to 0)	−0.2 (−0.5 to 0)	0 (−0.3 to 0.2)	0.76
Hypnotic use^c^	−1.0 (−1.5 to −0.5)	−0.8 (−1.3 to −0.2)	−0.3 (−0.9 to 0.4)	0.45
Daytime dysfunction^c^	−1.2 (−1.6 to −0.9)	−0.9 (−1.3 to −0.6)	−0.3 (−0.7 to 0.1)	0.18
Secondary outcome^b^				
ESS score at wk4, mean (95% CI)	4.4 (0 to 10.0)	4.6 (0 to 10.0)	−0.2 (−1.8 to 1.5)	0.86
Change at wk 4, ESS score, mean (95% CI)	−1.1 (−3.0 to 0.8)	−1.5 (−3.6 to 0.6)	0.4 (−1.4 to 2.2)	0.65
FFS score at wk4, mean (95% CI)	10.9 (4.1 to 19.9)	11.1 (5.1 to 18.9)	−0.2 (−2.5 to 2.0)	0.85
Change at wk 4, FFS score, mean (95%CI)	−3.4 (−5.8 to −1.0)	−3.6 (−6.0 to −1.3)	0.3 (−2.1 to 2.7)	0.82
SF-36 score at wk4, mean (95% CI)	576.8 (417.2 to 730.8)	520.8 (290.0 to 728.9)	60.0 (−8.9 to 120.9)	0.09
Change at wk 4, SF-36 score, mean (95% CI)	67.7 (14.1 to 121.2)	85.3 (14.3 to 156.2)	−17.6 (−70.2 to 35.0)	0.51
Physical function^c^	4.2 (−1.1 to 9.5)	5.0 (−3.2 to 13.2)	−0.8 (−7.0 to 5.4)	0.80
Role physical^c^	9.7 (−9.9 to 29.3)	10.2 (−12.9 to 33.2)	−0.5 (−22.5 to 21.6)	0.97
Bodily pain^c^	7.5 (−13.5 to 28.6)	13.5 (−8.3 to 35.4)	−6.0 (−25.6 to 13.6)	0.54
General health^c^	11.1 (1.9 to 20.4)	14.7 (5.5 to 23.9)	−3.6 (−11.8 to 4.6)	0.39
Vitality^c^	10.5 (1.6 to 19.3)	15.0 (5.8 to 24.2)	−4.5 (−11.9 to 2.8)	0.22
Social functioning^c^	2.9 (−8.2 to 14.0)	7.5 (−3.2 to 18.3)	−4.5 (−19.7 to 10.5)	0.55
Role emotional^c^	12.3 (3.5 to 21.1)	7.0 (−3.3 to 17.3)	5.2 (−3.8 to 12.2)	0.25
Mental health^c^	9.5 (1.1 to 17.9)	12.3 (1.9 to 22.8)	−2.8 (−10.4 to 4.7)	0.46
HAMD score at wk4, mean (95% CI)	6.1 (2.9 to 1.1)	7.6 (1 to 18.0)	−1.5 (−3.6 to 0.8)	0.20
Change at wk 4, HAMD score, mean (95% CI)	−5.8 (−8.0 to −3.6)	−7.7 (−10.5 to −4.8)	1.9 (−1.1 to 4.9)	0.21
HAMA score at wk4, mean (95% CI)	5.4 (0 to 11.0)	6.3 (0.1 to 15)	−0.9 (−3.1 to 1.3)	0.42
Change at wk 4, HAMA score, mean (95% CI)	−5.6 (−8.1 to −3.2)	−8.1 (−11.0 to −5.1)	2.4 (−0.2 to 5.1)	0.07

^a^All tests were 2-sided *P* value of less than 0.05 was considered significant. ^b^Missing data not imputed for outcomes analyses. ^c^Change at week 4 to baseline mean (95% CI).

## Data Availability

The data used to support the findings of this study are available from the corresponding author upon request.
