# Corrigendum to “Effect of Transcutaneous Vagus Nerve Stimulation at Auricular Concha for Insomnia: A Randomized Clinical Trial”

**DOI:** 10.1155/2020/2536573

**Published:** 2020-11-22

**Authors:** Yue Jiao, Xiao Guo, Man Luo, Suxia Li, Aihua Liu, Yufeng Zhao, Bin Zhao, Dequan Wang, Zaifang Li, Xiaojiao Zheng, Mozheng Wu, Peijing Rong

**Affiliations:** ^1^Institute of Acupuncture and Moxibustion, China Academy of Chinese Medical Sciences, Beijing 100700, China; ^2^National Institute on Drug Dependence, Peking University, Beijing 100191, China; ^3^Neurology Department, Xuanwu Hospital of Capital Medical University, Beijing 100053, China; ^4^Chinese Medicine Data Center, China Academy of Chinese Medical Sciences, Beijing 100700, China

In the article titled “Effect of Transcutaneous Vagus Nerve Stimulation at Auricular Concha for Insomnia: A Randomized Clinical Trial” [1], there was an error in Figure 1. The label in blue is to be corrected to “tn-VNS.” The authors confirm that this does not affect the results and conclusions of the article, and the corrected Figure 1 is as follows:

The authors also wish to correct the funding statement in the acknowledgements as follows:

## Figures and Tables

**Figure 1:**
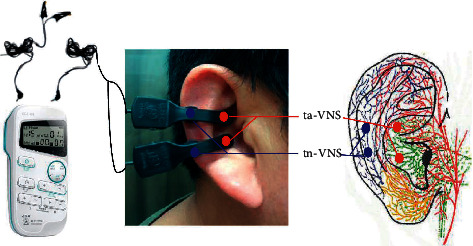
Stimulating regions of ta-VNS and tn-VNS. Red dots indicate regions of ta-VNS (ABVN: green color); blue dots indicate regions of tnVNS (LON: blue color).
